# Could Pretreatment Computed Tomography Imaging Accurately Predict the Pathological Diagnosis of Lymph Node Involvement in Thymic Epithelial Tumors?

**DOI:** 10.14740/wjon2746

**Published:** 2026-05-08

**Authors:** Hao-Yun Liu, Mei-Ci Chen, Jo-Yu Chen, Wen-Jeng Lee, Jang-Ming Lee

**Affiliations:** a Department of Medical Imaging, National Taiwan University Hospital Hsin-Chu Branch, Hsin-Chu, Taiwan, Republic of China; b National Taiwan University College of Medicine, Taipei, Taiwan, Republic of China; c Department of Medical Imaging, National Taiwan University, Taipei, Taiwan, Republic of China; d Department of Radiology, National Taiwan University, Taipei, Taiwan, Republic of China; e Department of Medical Imaging, National Taiwan University Hospital, Taipei, Taiwan, Republic of China; f Division of Thoracic Surgery, Department of Surgery, National Taiwan University Hospital, Taipei, Taiwan, Republic of China; g Department of Surgery, National Taiwan University College of Medicine, Taipei, Taiwan, Republic of China

**Keywords:** Thymic epithelial tumors, Lymph node metastasis, Computed tomography imaging, Sensitivity, Negative predictive value

## Abstract

**Background:**

Thymic epithelial tumors (TETs) are the most common primary malignancies of the anterior mediastinum. Initial treatment planning for TETs is based on staging determined through computed tomography (CT) imaging. This study aimed to demonstrate that pretreatment CT imaging can provide critical insights into lymph node (LN) metastasis in TETs, potentially guiding surgical decisions.

**Methods:**

Thirty patients with TETs treated at National Taiwan University Hospital from 2008 to 2023 were included. Three radiologists independently evaluated the pretreatment CT scans for LN metastasis, with findings validated against postoperative pathology reports. The study assessed the correlation between radiological and pathological LN findings, including criteria such as LN size, density, and morphology.

**Results:**

In a cohort of 30 TET patients, 43% were male, with an average age of just over 57 years. All patients underwent N1 LN dissection, and three also received N2 dissections. Pathological results confirmed LN metastasis in four cases (two thymoma B3, two thymic carcinoma), and 40% of patients were Masaoka-Koga stages III–IVb. Three experienced radiologists independently reviewed pretreatment CT scans and identified all four pathology-confirmed metastatic cases, yielding 100% sensitivity and negative predictive value (NPV) in this cohort. However, these findings should be interpreted cautiously because only four patients had pathologically confirmed LN metastasis.

**Conclusions:**

Pretreatment CT assessment may provide useful information for the evaluation of suspicious LN involvement in TETs and may assist preoperative planning. However, given the small number of metastatic cases, these findings should be considered preliminary, and further validation in larger, multicenter studies are required.

## Introduction

Thymic epithelial tumors (TETs), including thymoma, thymic carcinoma, and thymic neuroendocrine tumors, are the most common primary malignancies of the anterior mediastinum [1]. The initial treatment approach for TETs depends on staging based on computed tomography (CT) imaging. For resectable lesions identified on CT, surgery is the preferred treatment. Conversely, for unresectable lesions, neoadjuvant radiochemotherapy is selected as the primary intervention.

The Masaoka-Koga (M-K) staging system has been the widely accepted method for staging TETs since its initial publication [2]. In recent years, the International Thymic Malignancy Interest Group (ITMIG) and the International Association for the Study of Lung Cancer (IASLC) have advocated for the use of the tumor-node-metastasis (TNM) classification system for thymic malignancies [3–7]. However, due to the longstanding use of the M-K staging system, mediastinal lymph node (LN) dissection is less frequently practiced by thoracic surgeons. Although some surgeons emphasize the importance of LN dissection in treating thymic malignancies [8, 9], limited studies have explored this area.

Studies of LN involvement in TETs have primarily focused on pathological outcomes. Due to limited patient availability and the infrequent practice of LN dissection, research in this area has been restricted [10–16]. Although CT is routinely used for the preoperative staging of TETs, its specific diagnostic accuracy in predicting nodal metastasis remains unclear. When analyzing preoperative CT images, most thoracic surgeons and radiologists focus on the site and extent of tumor invasion, often overlooking LN metastasis evaluation. Therefore, this study aimed to investigate whether pretreatment CT imaging could provide useful information for predicting LN metastasis in TETs and thereby assist preoperative assessment and surgical planning.

## Materials and Methods

### Patient and data collection

Clinical records of patients who received medical treatment for TETs between January 2008 and December 2023 were collected from the National Taiwan University Hospital (NTUH) Medical Center. The inclusion criteria were as follows: 1) patient age between 15 and 80 years; 2) a confirmed final diagnosis of TETs; and 3) completion of the entire treatment course at NTUH. Exclusion criteria included: 1) absence of mediastinal LN findings in the postoperative pathology report; and 2) substantial missing or unverified medical data during collection. Most patients were followed in the NTUH Outpatient Department.

### Image analysis

The pretreatment CT scans were independently reviewed by three experienced thoracic radiologists, each blinded to the interpretations of the other readers and initially blinded to the final pathological results. In evaluating suspicious LN metastasis, the readers generally followed conventional thoracic oncologic imaging principles, considering nodal size, attenuation (CT density), and morphologic features, while also incorporating their clinical judgment based on routine practice. Because standardized CT criteria specific to LN metastasis in TETs are not currently established, no single fixed cutoff was used as the sole determinant of nodal positivity. The analysis was performed on a per-patient basis. No consensus adjudication was used for the primary analysis.

### Pathological analysis

The specimens were analyzed by pathologists with extensive experience, and diagnoses were established according to the World Health Organization (WHO) histological classification system [17]. Additionally, M-K staging was determined based on the extent of tumor invasion, as documented in the surgeon’s operative report. During mediastinal LN dissection, each LN was meticulously examined by the pathologists. All findings were systematically recorded in the NTUH database.

### Definitions

All enrolled patients were categorized according to the M-K staging classification based on preoperative CT imaging or perioperative evaluations, rather than final pathological reports [18, 19]. Patients with available WHO classifications were grouped by risk level: low-risk (types A, AB, and B1), moderate-risk (types B2 and B3), and high-risk (type C), following the categorization by Jeong et al [20]. Mediastinal LNs were designated as N1 or N2 groups per ITMIG guidelines [6]. Specifically, N1 nodes included anterior or peri-thymic nodes, while N2 nodes referred to deep intrathoracic or cervical nodes.

### Statistical analysis

All statistical analyses were performed using Predictive Analytics Software (PASW) version 25.0 (IBM Corp., Chicago, IL, USA). Continuous variables following a normal distribution are reported as mean ± standard deviation (SD). Diagnostic performance metrics were calculated on a per-patient basis. Sensitivity and specificity were derived through crosstab analysis. Positive predictive value (PPV) represents the proportion of cases where LN metastasis was identified by radiologists and subsequently confirmed in pathology reports. Negative predictive value (NPV) indicates the proportion of cases where radiologists reported no LN metastasis, which was corroborated by pathological findings. Overall accuracy was calculated as the ratio of true positive and true negative cases to the total of true positives, false negatives, true negatives, and false positives. Inter-rater agreement among the three radiologists was assessed using Fleiss’ kappa.

### Ethical compliance with human study

This study was conducted in accordance with the ethical standards of the institutional research committee and with the Declaration of Helsinki. This study protocol was approved by the NTUH Institutional Review Board (IRB), which granted a waiver of informed consent (Approval No. 202411101RINA).

## Results

### Baseline patient characteristics

Thirty patients participated in this study, with their baseline characteristics summarized in Table 1. The cohort included 43% males, with a mean age at surgery of just over 57 years. Three patients received chemotherapy or radiation therapy before operation. Nine patients underwent standard median sternotomy for tumor resection, whereas 19 received minimally invasive procedures. Each patient underwent N1 LN dissection, and three also had N2 LNs removed. Pathology reports identified mediastinal LN metastasis of TETs in four cases: two classified as thymoma type B3 and two as thymic carcinoma. Forty percent of the patients were classified as M-K stages III to IVb. Of the 30 patients, 21 patients were diagnosed with thymoma and nine patients with thymic carcinoma.

**Table 1:** Basic Characteristics of TETs Patients Receiving Operation and Mediastinal Lymph Node Dissection

	Patients (n = 30)	Range or %
Gender, male (%)	13	43.3%
Operation age, year	57.3 ± 10.9	38–74
Preoperative CT tumor size, cm	5.6 ± 2.7	2–11.5
Preoperative chemotherapy or radiation	3	10%
Operation method		
Median sternotomy	9	30%
Lateral thoracotomy	2	6.7%
VATS	14	46.7%
RATS	5	16.7%
Lymph node harvest		
N1	30	100%
N2	3	10%
Mediastinal lymph node metastasis	4	13.3%
M-K stage		
I	11	36.7%
IIa	4	13.3%
IIb	2	6.7%
III	7	23.3%
IVa	1	3.3%
IVb	4	13.3%
WHO histotype		
A + AB + B1 (low risk)	11	36.7%
B2 + B3 (moderate risk)	9	30%
Micronodular thymoma with lymphoid stroma	1	3.3%
Carcinoma	9	30%

TETs: thymic epithelial tumors; CT: computed tomography; VATS: video-assisted thoracoscopic surgery; RATS: robotic-assisted thoracic surgery; WHO: World Health Organization; M-K: Masaoka-Koga.

### Evaluation of CT image

Three radiologists participated in this study, each with substantial experience: Radiologist A with 26 years, Radiologist B with 10 years, and Radiologist C with 9 years. All were attending physicians who assessed mediastinal LN metastasis in patients with TETs using pretreatment CT imaging. The outcomes of their evaluations are detailed in Table 2. As shown, all three radiologists identified all four pathology-confirmed metastatic cases, yielding a sensitivity of 100% in this cohort. Moreover, each radiologist’s NPV was also 100%. However, these findings should be interpreted with caution because only four patients had pathologically confirmed LN metastasis.

**Table 2:** Evaluation of Sensitivity, Specificity, and Predictive Values by Identifying TETs Lymph Node Metastasis From CT Image

	TP	FP	TN	FN	Sensitivity	Specificity	PPV	NPV	Accuracy
TETs (n = 30)									
Radiologist A	4	2	24	0	100%	92.3%	66.7%	100%	93.3%
Radiologist B	4	10	16	0	100%	61.5%	28.6%	100%	66.7%
Radiologist C	4	6	20	0	100%	76.9%	40%	100%	80%

Mediastinal lymph node metastasis was recorded in four patients with TETs in the pathology reports. TETs: thymic epithelial tumors; CT: computed tomography; TP: true positive; FP: false positive; TN: true negative; FN: false negative; PPV: positive predictive value; NPV: negative predictive value.

Furthermore, the overall inter-rater agreement among the three radiologists was fair (Fleiss’ kappa = 0.35), suggesting that a certain degree of variability remained among readers in the CT-based assessment of LN metastasis.

## Discussion

The main finding of this study was that none of the four pathology-confirmed metastatic cases were missed by any of the three radiologists in this cohort. However, because only four patients had pathologically confirmed nodal metastasis, these results should be regarded as preliminary and interpreted cautiously.

The criteria for evaluating LN metastasis on CT imaging were based on LN size and density. All three radiologists demonstrated high sensitivity and NPV; however, only two of the three achieved specificities above 75%. In contrast to Radiologists A and C, Radiologist B showed a higher rate of false positives. Upon review, it was determined that, besides assessing size and density, Radiologist B rigorously evaluated LN morphology, including shape and contour. Radiologist B identified even small, low-density LNs with abnormal morphology as metastases. Radiologists A and C used similar evaluation criteria but applied a more relaxed approach to morphological features, informed by their understanding of the lower rate of mediastinal LN metastasis in TETs patients.

**Figure 1:**
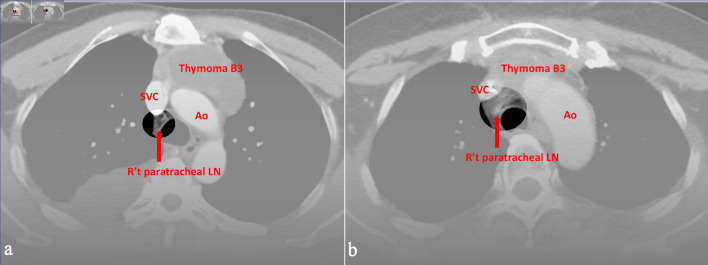
Preoperative CT images of (a) a 52-year-old male and (b) a 49-year-old female with World Health Organization (WHO) classification B3. All three radiologists evaluated right upper paratracheal LN metastasis, and the pathological report proved the positive findings. Ao: aorta; SVC: superior vena cava; CT: computed tomography; LN: lymph node.

**Figure 2:**
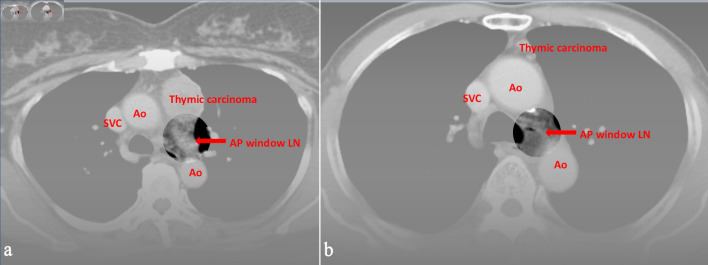
Preoperative CT images of (a) a 59-year-old female and (b) a 74-year-old male with World Health Organization (WHO) classification thymic carcinoma. All three radiologists evaluated LN metastasis in the aortopulmonary window, and the pathological report proved the positive findings. Ao: aorta; SVC: superior vena cava; CT: computed tomography; LN: lymph node.

Compared with lung or esophageal cancer, TETs are rare. There is limited experience with image evaluation by the radiologists within individual hospitals. The most commonly used and convenient method for assessing LN metastasis was LN enlargement. Figures 1 and 2 provided the preoperative CT images in which the evaluations of all three radiologists are consistent with the pathological reports. As shown in the figures, these metastasis LNs were differed from normal LNs, although not all were LN enlargement. The study by Shen et al [21] showed low sensitivity and high specificity judged by LN enlargement from CT image, which differs from this study outcome. This means a higher bias in the evaluation of LN metastasis in TETs. However, if the judgment criteria are made more stringent, surgeon might harvest more non-metastatic LNs. Depending on the limited data, it would be hard to make effective suggestions in this study. At the very least, preoperative discussion between surgeons and radiologists regarding suspicious mediastinal LNs on CT may help improve awareness of potential nodal involvement and may facilitate more careful intraoperative assessment. But compared with no intervention, from the perspective of removing LNs, it can provide additional research materials for subsequent researchers.

Among the 30 patients, three underwent chemotherapy or radiotherapy prior to surgery. Of the four patients with LN metastases, one had a history of preoperative chemoradiotherapy. Notably, two patients without LN metastases in the postoperative pathology report had received chemoradiotherapy before surgery. In these two cases, pretreatment CT scans interpreted by radiologists suggested the presence of LN metastases. However, the pathology report reflects only the status of LN metastasis at the time of surgery and does not provide information on whether LNs were previously invaded by TETs and subsequently responded to chemoradiotherapy. This raises the possibility that the radiologist’s assessment of LN metastasis on CT may not have been falsely positive as frequently as assumed.

Fluorine-18 fluorodeoxyglucose (18F-FDG) positron emission tomography (PET)-CT has emerged as a diagnostic tool for clinical staging of intrathoracic malignancies, such as lung and esophageal cancer. Although FDG PET-CT is useful for diagnosing thymic carcinoma, it is less useful for thymoma because thymomas are not FDG-avid [22, 23]. As a result, FDG PET/CT does not have a routine role in staging of thymoma. In real-world practice in Taiwan, CT is typically the first-line imaging modality for preoperative evaluation due to its lower cost and convenience, whereas FDG PET/CT is not routinely performed. FDG PET/CT was reserved for patients diagnosed with thymic carcinoma, which was considered advanced disease based on initial CT findings, to guide subsequent treatment planning.

Table 3 [10–16] lists the clearer studies on LN metastasis in TETs. The study by Kondo et al was the first large-scale one that mentioned LN dissection in TETs. The incidence of LN metastasis was 1.8% in thymoma, and 26.8% in thymic carcinoma [10]. Weksler et al used the Surveillance, Epidemiology, and End Results (SEER) database to analyze the incidence of LN metastasis, which was higher than that reported by Kondo et al [13, 14]. Most studies, especially those from Asian populations, show that LN metastasis in thymoma is rare, most of which does not exceed 10%. The LN metastasis rate of thymic carcinoma is about 25%. There may be some racial differences; however, more data are needed for analysis. Initially, these published researches showed the map of a lower LN metastasis in both thymoma and thymic carcinoma. This initial thought might stop the surgeons arranging LN dissection during the operation. So, Ahmad et al advocated for LN dissection in TETs for further study [8]. Even within the ITMIG database, the current TNM staging for thymic malignancy defined N1/2 as stage IV because of lower patient numbers for detail analysis. More information on LN dissection in TETs was needed for further stage system.

**Table 3:** Previous Studies for TETs LN Metastasis

	Years of inclusion	Lymph node harvest patient numbers	Lymph node metastasis rate
Kondo et al, 2003, [10]	1990–1994	Thy: 1,064	1.8%
		TC: 183	26.8%
Park et al, 2013 [11]	1995–2010	TC: 26	23.1%
Weissferdt et al, 2012 [12]	1985–2011	TC: 30	36.7%
Weksler et al, 2015 [13]	1988–2009	Thy: 442	13.3%
Weksler et al, 2015 [14]	1988–2011	TC: 176	64.1%
		NETT: 53	35.9%
Hwang et al, 2016 [15]	1996–2011	Thy: 99	5.1%
		TC: 32	25.0%
Fang et al, 2018 [16]	2014–2016	Thy: 243	2.1%
		TC: 24	25.0%
		NETT: 8	50.0%
Current study	2008–2023	Thy: 21	9.5%
		TC: 9	22.2%

TETs: thymic epithelial tumors; LN: lymph node; Thy: thymoma; TC: thymic carcinoma; NETT: neuroendocrine thymic tumor.

The limited number of studies focusing on LN might be attributed to the fact that relatively few thoracic surgeons routinely perform LN dissection during surgery. This is also related to the Masaoka stage, which has led most surgeons and radiologists to focus primarily on the extent of tumor involvement based on pretreatment CT imaging, often overlooking the possibility of LN metastasis. This has led surgeons to pay more attention to tumor resection rather than LN dissection. Even if they were aware of the importance of LN dissection, they often forgot to do so. Addressing this issue requires stronger institutional coordination and greater advocacy efforts. It is only by recognizing the significance of LN involvement that more data can be made available for analysis in the future.

This study has several limitations worth noting. First, due to the rarity of thymic tumors, our sample size was relatively small, which may have limited statistical power. Most importantly, only four patients had pathologically confirmed LN metastasis; therefore, the observed 100% sensitivity and NPV should be considered preliminary findings and interpreted with caution. Second, as a retrospective single-center study with a long accrual period, this study may be subject to selection bias and limited generalizability. Third, the heterogeneity of the patient cohort, encompassing a range of tumor stages and histological subtypes, may have affected result interpretation. Fourth, technical limitations included variations in CT scanner specifications and possible changes in imaging protocols over time, including differences in slice thickness and reconstruction intervals across examinations. Fifth, radiological assessment of LN could vary, as different radiologists may interpret the same CT images differently, and even the same radiologist might reach varying conclusions on separate occasions. Additional challenges arose in evaluating LN in certain anatomical regions, where limited visibility or complex surrounding structures added complexity. The absence of standardized criteria for LN assessment also represents a significant study limitation; currently, there are no universally accepted imaging criteria for determining LN positivity in thymic tumors, particularly with respect to optimal cut-off values for LN size, morphology, or enhancement on CT. This lack of standardization could lead to inconsistent interpretations across different clinical settings. In addition, because of the retrospective design and the long study period, original histologic images from some pathology-confirmed cases were not available for retrieval or suitable publication-quality presentation, which limited complete radiologic-pathologic correlation in this study. Lastly, our results may not entirely capture the nuances of real-world clinical decision-making, where radiologists and clinicians often integrate multiple factors beyond imaging findings, including clinical symptoms, laboratory data, and additional imaging modalities. Given the diverse biological behavior and imaging characteristics of different thymic tumor subtypes, these findings may not be fully applicable across all histological variants, potentially limiting their clinical relevance in specific patient subgroups.

### Conclusions

This study presents a single-center experience in evaluating LN metastasis in TETs through pretreatment CT imaging. Our findings suggest that suspicious mediastinal LNs on pretreatment CT may warrant careful nodal assessment and consideration during surgical planning. However, given the limited number of metastatic cases, these findings should be regarded as preliminary, and further validation in larger, multicenter studies is required. This study aims to raise awareness among thoracic surgeons about the importance of LN dissection in TETs and provide detailed data for future research. This approach could contribute to the development of a more precise staging system, ultimately leading to more appropriate treatment strategies for patients with TETs.

## Data Availability

The data supporting the findings of this study are available from the corresponding author upon reasonable request.

## Abbreviations

TETs: thymic epithelial tumors
CT: computed tomography
M-K: Masaoka-Koga
ITMIG: International Thymic Malignancy Interest Group
IASLC: International Association for the Study of Lung Cancer
LN: lymph node
PPV: positive predictive value
NPV: negative predictive value

## References

[1] Morgenthaler TI, Brown LR, Colby TV, Harper CM, Coles DT (1993). Thymoma. Mayo Clin Proc.

[2] Masaoka A, Monden Y, Nakahara K, Tanioka T (1981). Follow-up study of thymomas with special reference to their clinical stages. Cancer.

[3] Detterbeck FC, Asamura H, Crowley J, Falkson C, Giaccone G, Giroux D, Huang J (2013). The IASLC/ITMIG thymic malignancies staging project: development of a stage classification for thymic malignancies. J Thorac Oncol.

[4] Detterbeck FC, Stratton K, Giroux D, Asamura H, Crowley J, Falkson C, Filosso PL (2014). The IASLC/ITMIG thymic epithelial tumors staging project: proposal for an evidence-based stage classification system for the forthcoming (8th) edition of the TNM classification of malignant tumors. J Thorac Oncol.

[5] Nicholson AG, Detterbeck FC, Marino M, Kim J, Stratton K, Giroux D, Asamura H (2014). The IASLC/ITMIG Thymic Epithelial Tumors Staging Project: proposals for the T Component for the forthcoming (8th) edition of the TNM classification of malignant tumors. J Thorac Oncol.

[6] Kondo K, Van Schil P, Detterbeck FC, Okumura M, Stratton K, Giroux D, Asamura H (2014). The IASLC/ITMIG thymic epithelial tumors staging project: proposals for the N and M components for the forthcoming (8th) edition of the TNM classification of malignant tumors. J Thorac Oncol.

[7] Bhora FY, Chen DJ, Detterbeck FC, Asamura H, Falkson C, Filosso PL, Giaccone G (2014). The ITMIG/IASLC thymic epithelial tumors staging project: a proposed lymph node map for thymic epithelial tumors in the forthcoming 8th edition of the TNM classification of malignant tumors. J Thorac Oncol.

[8] Ahmad U, Raja S (2017). Lymph node metastases in thymic tumors: The more we know, the less we know. J Thorac Cardiovasc Surg.

[9] Clermidy H, Maury JM, Collaud S, Drevet G, Ginoux M, Chalabreysse L, Mornex F (2021). Lymph node dissection in thymoma: Is it worth it?. Lung Cancer.

[10] Kondo K, Monden Y (2003). Lymphogenous and hematogenous metastasis of thymic epithelial tumors. Ann Thorac Surg.

[11] Park IK, Kim YT, Jeon JH, Kim HS, Hwang Y, Seong YW, Kang CH (2013). Importance of lymph node dissection in thymic carcinoma. Ann Thorac Surg.

[12] Weissferdt A, Moran CA (2012). Thymic carcinoma, part 2: a clinicopathologic correlation of 33 cases with a proposed staging system. Am J Clin Pathol.

[13] Weksler B, Pennathur A, Sullivan JL, Nason KS (2015). Resection of thymoma should include nodal sampling. J Thorac Cardiovasc Surg.

[14] Weksler B, Holden A, Sullivan JL (2015). Impact of positive nodal metastases in patients with thymic carcinoma and thymic neuroendocrine tumors. J Thorac Oncol.

[15] Hwang Y, Park IK, Park S, Kim ER, Kang CH, Kim YT (2016). Lymph node dissection in thymic malignancies: implication of the ITMIG lymph node map, TNM Stage Classification, and Recommendations. J Thorac Oncol.

[16] Fang W, Wang Y, Pang L, Gu Z, Wei Y, Liu Y, Zhang P (2018). Lymph node metastasis in thymic malignancies: a Chinese multicenter prospective observational study. J Thorac Cardiovasc Surg.

[17] Marx A, Strobel P, Badve SS, Chalabreysse L, Chan JK, Chen G, de Leval L (2014). ITMIG consensus statement on the use of the WHO histological classification of thymoma and thymic carcinoma: refined definitions, histological criteria, and reporting. J Thorac Oncol.

[18] Fukui T, Fukumoto K, Okasaka T, Kawaguchi K, Nakamura S, Hakiri S, Ozeki N (2016). Prognostic impact of tumour size in completely resected thymic epithelial tumours. Eur J Cardiothorac Surg.

[19] Okumura M, Yoshino I, Yano M, Watanabe SI, Tsuboi M, Yoshida K, Date H (2019). Tumour size determines both recurrence-free survival and disease-specific survival after surgical treatment for thymoma. Eur J Cardiothorac Surg.

[20] Jeong YJ, Lee KS, Kim J, Shim YM, Han J, Kwon OJ (2004). Does CT of thymic epithelial tumors enable us to differentiate histologic subtypes and predict prognosis?. AJR Am J Roentgenol.

[21] Shen Y, Gu Z, Ye J, Mao T, Fang W, Chen W (2016). CT staging and preoperative assessment of resectability for thymic epithelial tumors. J Thorac Dis.

[22] Benveniste MFK, Betancourt Cuellar SL, Carter BW, Strange CD, Marom EM (2021). Thymic epithelial neoplasms: tumor-node-metastasis staging. Radiol Clin North Am.

[23] Strange CD, Ahuja J, Shroff GS, Truong MT, Marom EM (2021). Imaging evaluation of thymoma and thymic carcinoma. Front Oncol.

